# Haplotypes of *FOXP3* genetic variants are associated with susceptibility, autoantibodies, and TGF-β1 in patients with systemic lupus erythematosus

**DOI:** 10.1038/s41598-021-84832-3

**Published:** 2021-03-08

**Authors:** Nicole Perugini Stadtlober, Tamires Flauzino, Lorena Flor da Rosa Franchi Santos, Tatiana Mayumi Veiga Iriyoda, Neide Tomimura Costa, Marcell Alysson Batisti Lozovoy, Isaias Dichi, Edna Maria Vissoci Reiche, Andréa Name Colado Simão

**Affiliations:** 1 Laboratory of Research in Applied Immunology, State University of Londrina, Londrina, PR Brazil; 2 Department of Rheumatology, Pontifical Catholic University of Paraná, Londrina, PR Brazil; 3 Department of Rheumatology, State University of Londrina, Londrina, PR Brazil; 4 Department of Pathology, Clinical Analysis and Toxicology, Laboratory of Research in Applied Immunology, Health Sciences Center, University Hospital, State University of Londrina, Av. Robert Koch 60, Londrina, Paraná, CEP 86038-440 Brazil; 5 Department of Internal Medicine, Laboratory of Research in Applied Immunology, State University of Londrina, Londrina, PR Brazil

**Keywords:** Genetics, Immunology, Molecular biology, Rheumatology

## Abstract

The aim of this study was to evaluate the association of rs2232365 (-924 G > A) and rs3761548 (-3279 C > A) *FOXP3* variants with systemic lupus erythematosus (SLE) susceptibility, TGF-β1 plasma levels, autoantibodies, and LN nephritis, and SLE disease activity index (SLEDAI). The study included 196 SLE female patients and 157 female controls. *FOXP3* variants were determined with polymerase chain reaction-restriction fragment length polymorphism (PCR–RFLP). Plasma levels of TGF-β1 were determined using immunofluorimetric assay. The AA genotype [OR: 2.650, CI 95%(1.070–6.564), *p* = 0.035] and A allele [OR: 2.644, CI 95%(1.104–6.333), *p* = 0.029] were associated with SLE diagnosis in the -3279 C > A. The A/A haplotype was associated with SLE [OR: 3.729, CI 95%(1.006–13.820), *p* = 0.049]. GCGC haplotype patients had higher TGF-β1 levels (*p* = 0.012) than other haplotypes. Patients with -924 AA genotype showed higher frequency of anti-dsDNA (*p* = 0.012) and anti-U1RNP (*p* = 0.036). The A/C haplotype had higher SLEDAI score [OR: 1.119, CI 95%(1.015–1.234), *p* = 0.024] and ACAC haplotype higher frequency of anti-dsDNA [OR: 3.026, CI 95%(1.062–8.624), *p* = 0.038], anti-U1RNP [OR: 5.649, CI 95%(1.199–26.610), *p* = 0.029] and nephritis [OR: 2.501, CI 95%(1.004–6.229), *p* = 0.049]. Our data demonstrate that the G/C haplotype provides protection for SLE. While the presence of allele A of both variants could favor autoimmunity, disease activity, and LN.

## Introduction

Systemic lupus erythematosus (SLE) is an autoimmune inflammatory disease, characterized by dysregulated innate and adaptive immune responses with multiple organ damage^1^. Disease manifestation is very heterogeneous with different characteristics, ranging from laboratory abnormalities to multiorgan failure^2^. Lupus nephritis (LN) is a severe manifestation of SLE, affecting approximately 40–70% of patients and contributes substantially to disease morbidity and mortality^3^.

The etiology of SLE is not completely elucidated but involves an interaction between genetic, hormonal and environmental factors^4–6^. The pathophysiology of SLE is complex and characterized by the production of a large amount of autoantibodies that act against nuclear cells structures and promote inflammation and tissue damage^7^. The generation of autoantibodies is a result of various immunological changes, including inappropriate regulation of B and T cells, loss of immune tolerance, and defective clearance of apoptotic cells and immune complexes^8^.

The regulatory CD4^+^CD25^+^ T (Treg) cells are a subset of CD4^+^ T cells that plays a crucial role in suppression of the immune response^9^ by TGF-β1 and IL-10 production^10^. The majority of Treg cells arise during thymic T-cells maturation and are characterized by high constitutive expression of the IL-2 receptor alfa chain (CD25) and the transcription factor forkhead box protein 3 (FoxP3)^11,12^. The FoxP3 belongs to the forkhead/winged-helix family of transcription factors and it is considered the master regulator of Treg cells development and function ^13,14^. *FOXP3* gene is located on chromosome Xp11.23 with a highly conserved forkhead DNA-binding domain. An expression of this gene is essential for CD4^+^ T cells differentiation into CD4^+^CD25^+^ Treg cells^15^. Treg cells depletion has also been associated with the pathogenesis, severity, and periods of disease activity of SLE^16–18^.

Different single nucleotide variants (SNVs) have been described in the promoter region of *FOXP3*, which can affect the expression of FoxP3 and impair the Treg cells differentiation and function^19^. The -924 G > A (rs2232365) and -3279 C > A (rs3761548) *FOXP3* variant were associated with the susceptibility and prognosis of various autoimmune diseases, such as rheumatoid arthritis^20,21^, multiple sclerosis^22^, and SLE^8^.

Recently, our group demonstrated that -3279 C > A of *FOXP3* variant was associated to multiple sclerosis diagnosis in female patients^22^. Regarding the other *FOXP3* variant, the -924 G > A was evaluated in some autoimmunity diseases^23–25^ but not in SLE. Thus, we hypothesized that *FOXP3* variants could influence Treg cells function, by the inhibition of TGF-β1 production, and promoting autoantibodies and disease activity in SLE patients. Therefore, the aim of this study was to evaluate the -924 G > A (rs2232365) and -3279 C > A (rs3761548) *FOXP3* variants and their association with SLE susceptibility, TGF-β1 plasma levels, presence of autoantibodies and LN, and SLE disease activity index (SLEDAI) in SLE patients.

## Subjects and methods

### Subjects

This is a case–control study that included 353 adult participants. Among them, 196 were SLE female patients, consecutively recruited during the 2016 to 2018 period of the Rheumatology Outpatient Clinic of the University Hospital of Londrina-Paraná/Brazil. The SLE diagnosis was established according to the American College of Rheumatology (ACR) criteria^26^. The SLEDAI-2 K score was used to determine disease activity and values ​​ ≥ 6 were used as a parameter to classify moderate and high disease activity and < 6 to inactive and mild disease activity^27,28^. LN was reported based on medical history or by the presence of proteinuria (≥ 0.5 g/24 h) and/or hematuria or pathological finding in the urine sediment, with or without an increase in creatinine serum levels^29^. All patients had LN confirmed by biopsy. As controls, 157 healthy female were selected from blood donors of the Regional Blood Center of Londrina. Patients and controls were matched by age, ethnicity and body mass index (BMI).

Inclusion criteria was age between 18 and 69 years old. The exclusion criteria were the presence of other inflammatory, infectious, autoimmune and neoplastic diseases. Information about lifestyle, medical history, treatment and blood collection were obtained at the time of inclusion in the study. All participants gave written informed consent, and the study protocol was fully approved by the Institutional Research Ethics Committees of State University of Londrina, Paraná, Brazil (CAAE: 01865212.0.0000.5231).

### Anthropometric measurements

Body weight was measured to the nearest 0.1 kg using electronic scales, with individuals wearing light clothing, but no shoes, in the morning; height was measured to the nearest 0.1 cm by using a stadiometer. BMI was calculated as weight (kg) divided by height (m) squared.

### Blood collection and immunological biomarkers

After fasting for 12 h, venous blood samples were obtained with ethylenediaminetetraacetic acid (EDTA) as anticoagulant and without anticoagulant. Further, whole blood was centrifuged at 3000 rpm for 15 min and serum, plasma and buffy-coat were separated, divided into aliquots, and stored at − 80 °C until use. Serum levels of complement, C3 and C4 were assessed by turbidimetry (C800, Abbott Laboratory, Abbott Park, IL, USA).

Antinuclear antibodies (ANA) were quantified using indirect immunofluorescence with HEp2 cells as a substrate (IFI-ANA-HEp2-IgG; VIRO-IMMUN Labor Diagnostika, GmbH, Oberursel, Germany) and were considered significant when titers ≥ 1:80. Anti-double stranded DNA (Anti-dsDNA), anti-nucleosome, anti-Smith (anti-SM), anti-U1 ribonucleoprotein (anti-U1RNP) antibodies were quantified by antibody enzyme immunoassay (ELISA, Orgentec Diagnostika, GmbH, Germany) and were considered positive when results ≥ 20 IU/mL.

TGF-β1 plasma levels were determined using microspheres immunofluorimetric assay (ProcartaPlex by Thermo Fisher Scientific, Vienna, Austria) for Luminex platform (MAGPIX, Luminex Corp., Austin, TX, USA). All analyzes were performed according to the manufacturer's instructions.

### Genomic DNA extraction

Genomic DNA was extracted from a buffy-coat of peripheral blood cells using a resin column procedure (Biopur, Biometrix Diagnostika, Curitiba, Brazil), following the manufacturer’s recommendations. The DNA concentration was measured with a NanoDrop 2000c spectrophotometer (ThermoScientific, Waltman, MA, USA) at 260 nm and purity was assessed by measuring the 260/280 nm ratio.

### FOXP3 genetic variant genotyping

Two SNVs in the promoter region of the *FOXP3* were genotyped: -924 G > A (rs2232365) at position 49259426 and -3279 C > A (rs3761548) at position 49261784 according to listed in the international database and to GenBank accession number (NG_007392.1).

Polymerase chain reaction-restriction fragment length polymorphism (PCR–RFLP) analysis was carried out using peripheral blood genomic DNA to detect the rs2232365 and rs3761548 SNVs, as previously reported by^30^ with some modifications. For rs2232365 genotyping, the following primers were used: 5´-AGGAGAAGGAGTGG GCATTT-3´ (forward) and 5´-GTGAGTGGAGGAGCTGA GG-3´ (reverse)^20^. For rs3761548 genotyping was performed with the following primers: 5′-GGCAGAGTTGAAATCCAAGC-3′ (forward) and 5′-CAACGTG TGAGAAGGCAGAA-3′ (reverse)^31^. The PCR was performed in a thermal cycler (Applied Biosystems VERITI 96-well Thermal Cycler, Life Technologies, Foster City, CA, USA) with a negative control (without a DNA sample).

PCR products of rs2232365 [249 base pairs (bp)] were digested overnight at 37ºC with *Esp*3I restriction endonuclease (ANZA, Invitrogen, Life Technologies, Carlsbad, CA, USA), generating two fragments of 132 bp and 117 bp corresponding to G allele, while the A allele that did not undergo enzymatic cleavage and remained with 249 bp. PCR products of rs3761548 (155 bp) were digested with *Pst*I restriction endonuclease (ANZA, Invitrogen, Life Technologies, Carlsbad, CA, USA), which generated two fragments, 80 bp and 75 bp, that correspond to C allele, while the A allele remained with 155 bp. All PCR–RFLP products were analyzed using 10% polyacrylamide gel and stained with silver nitrate.

### Statistical analysis

Categorical data were evaluated by chi-square (*χ*^2^) test and expressed as absolute number (n) and percentage (%). The odds ratio (OR) and 95% confidence interval (95% CI) were calculated. Continuous data were evaluated by Mann–Whitney test and expressed as median and percentile range (25%–75%). The p value was adjusted for multiple variables (age, ethnicity, BMI, and treatment) by binary logistic or multinomial regression test, when appropriate. Hardy–Weinberg equilibrium (HWE) and the estimation of pairwise linkage disequilibrium (LD) were performed in Haploview software version 4.2. LD between the specified SNVs was provided by describing D and r-squared value. Inference of recombination sites between *FOXP3* alleles were determined using the PHASE software version 2.1.1 by assigning each haplotype with maximum probability^32,33^. All statistical analyzes were performed with SPSS for Windows, version 22.0 (SPSS 31 Inc., CHIGADO, IL, USA) and statistical significance was set at *p* < 0.05.

### Ethical approval

This study was conducted after approval by the Institutional Research Ethics Committees of University of Londrina, Paraná, Brazil (CAAE: 01,865,212.0.0000.5231). All procedures performed in studies involving human participants were in accordance with the ethical standards of the institutional and/or national research committee and with the 1964 Helsinki declaration and its later amendments or comparable ethical standards.

## Results

Table 1 shows the baseline data of female patients with SLE. The median disease duration was 10 years (4–15), the mode of ANA titers was 1:320 (1:80–1:5120) and the median of SLEDAI score was 2 (1–6). Based on SLEDAI values, most of patients (81.3%) had inactive disease. The median of C3 was 112.5 mg/dL (92–133) and C4 20.1 mg/dL (13.5–25.9). The median of anti-nucleosome levels was 53.74 IU/mL (19.07–138.74), 58.2% had positive anti-dsDNA antibodies, 23.1% had positive anti-SM antibodies and 43.2% had positive anti-U1RNP antibodies. In addition, 87 (46.5%) patients had LN. Regarding treatment, 91.0% of patients used corticosteroids and the median dose was 8 (5–20) mg/day. Antimalarial was used by 74.1% patients, immunosuppressive by 44.4%, and mycophenolate by 22.8%.

**Table 1:** Clinical and laboratory parameters of patients with systemic lupus erythematosus (SLE).

Characteristics	N = 196
Disease duration (years)	10 (4–15)
SLEDAI	2 (1–6)
≥ 6	36 (18.7)
< 6	157 (81.3)
ANA (titer)	1:320 (1:80–1:5120)
C3 (mg/dL)	112.5 (92.0–133.0)
C4 (mg/dL)	20.1 (13.5–25.9)
Anti-nucleosome (IU/mL)	53.74 (19.07–138.74)
Anti-dsDNA (IU/mL)	28.44 (8.00–72.27)
Positive	107 (58.2)
Negative	77 (41.8)
Anti-SM (IU/mL)	6.43 (3.79–17.63)
Positive	30 (23.1)
Negative	100 (76.9)
Anti-U1RNP (IU/mL)	13.09 (5.21–83.75)
Positive	54 (43.2)
Negative	71 (56.8)
Lupus nephritis	87 (46.5)
Treatment	
Prednisone	172 (91)
Prednisone (mg/day)	8 (5–20)
Antimalarials	140 (74.1)
Mycophenolate	43 (22.8)
Immunosuppressive	84 (44.4)

Data were expressed by median and percentile (25–75%) or absolute number (n) and percentage (%). ANA: semi quantitative values expressed in titers and analyzed as mode. SLEDAI: systemic lupus erythematosus disease activity index; ANA: antinuclear antibodies; C3: complement 3; C4: complement 4; Anti-dsDNA: anti-double-stranded DNA; Anti-SM; anti-Smith; Anti-U1RNP: anti-U1 ribonucleoprotein.

The two SNVs of *FOXP3* (rs2232365/rs3761548) were genotyped in 196 SLE patients and 157 healthy controls and divided into three genetic models (dominant, codominant, and recessive) to assess the association with SLE susceptibility (Table 2). As expected, patients and controls did not differ in age, ethnicity and BMI (data not shown). However, we control the possible interference of these variables in the analysis.

**Table 2:** Distribution of *FOXP3* -924 G > A (rs2232365) and -3279 C > A (rs3761548) genotypes and allelic frequencies among patients with systemic lupus erythematosus (SLE) and controls.

Genetic model	Controls (n = 157^1^)	SLE (n = 193^2^)	OR (95% CI)	*p* value*
**rs2232365** **-924 G > A**				
Allelic				
G	169 (53.8)	203 (52.6)	Reference	
A	145 (46.2)	183 (47.4)	1.344 (0.808–2.237)	0.255
Codominant				
GG	42 (26.8)	45 (23.3)	Reference	
GA	85 (54.1)	113 (58.5)	1.379 (0.812–2.342)	0.234
AA	30 (19.1)	35 (18.1)	1.243 (0.635–2.434)	0.525
Dominant				
GG	42 (26.8)	45 (23.3)	Reference	
GA + AA	115 (73.2)	148 (76.7)	1.317 (0.792–2.190)	0.288
Recessive				
GG + GA	127 (80.9)	158 (81.9)	Reference	
AA	30 (19.1)	35 (18.1)	0.993 (0.567–1.739)	0.981

Genetic Model	Controls (n = 155^1^)	SLE (n = 196^2^)	OR (95% CI)	*p* value*
**rs3761548** **-3279 C > A**				
Allelic				
C	217 (70.0)	260 (66.3)	Reference	
A	93 (30.0)	132 (33.7)	2.644 (1.104–6.333)	**0.029**
Codominant				
CC	70 (45.2)	85 (43.4)	Reference	
CA	77 (49.7)	90 (45.9)	1.004 (0.632–1.596)	0.986
AA	8 (5.2)	21 (10.7)	2.650 (1.070–6.564)	**0.035**
Dominant				
CC	70 (45.2)	85 (43.4)	Reference	
CA + AA	85 (54.8)	111 (56.6)	1.150 (0.735–1.798)	0.540
Recessive				
CC + CA	147 (94.8)	175 (89.3)	Reference	
AA	8 (5.2)	21 (10.7)	2.644 (1.104–6.333)	**0.029**

*χ*^2^: results of analyses of contingency tables. Data were expressed as absolute number (n) and percentage (%). Bold values represent statistically significant values. *Adjusted by age and ethnicity.

^1^Hardy–Weinberg equilibrium *χ*^2^: rs3761548 = 5.18, *p* < 0.05; rs2232365 = 1.25, *p* > 0.05.

^2^Hardy–Weinberg equilibrium *χ*^2^: rs3761548 = 0.15, *p* > 0.05; rs2232365 = 5.85, *p* < 0.05.

The HWE of rs3761548 and rs2232365 in SLE patients and healthy controls was assessed and genotype frequencies presented divergence from HWE (*χ*^2^ test; *p* < 0.05), excepted by rs3761548 in SLE group and rs2232365 in control group (*χ*^2^ test; *p* > 0.05). In the -924 G > A (rs2232365) variant, the frequency of the GG, GA, and AA genotypes (codominant, dominant, and recessive genetic models) did not differ between SLE patients and controls (*p* > 0.05). No significant associations were found in the allele frequencies (OR 1.344, 95% CI 0.808–2.237, *p* = 0.255) (Table 2).

Regarding the -3279 C > A (rs3761548) variant, the results demonstrated that the frequency of CC, CA, and AA genotypes (codominant genetic model) differed between SLE patients and controls. The AA genotype was directly associated with SLE diagnosis (OR 2.650, 95% CI 1.070–6.564, *p* = 0.035). When the dominant genetic model was evaluated in SLE and controls groups, no significant association was observed in the frequency of CA + AA genotypes vs CC (*p* > 0.05). In the recessive genetic model, the presence of AA genotype was higher in SLE patients when compared to controls, 21 (10.7%) versus 8 (5.2%) respectively (OR 2.644, 95% CI 1.104–6.333, *p* = 0.029). Furthermore, the allelic model showed that the presence of A allele was associated with SLE diagnosis (OR 2.644, 95% CI 1.104–6.333, *p* = 0.029). All data were adjusted by ethnicity and age.

Four possible haplotype combinations with rs2232365 and rs3761548 were investigated in our study: A/C, A/A, G/A, and G/C. The LD between *FOXP3* rs2232365 and rs3761548 showed that those SNVs are not good surrogate markers for each other (D′ = 0.796; r^2^ = 0.265). Therefore, it is important to assess their combined effects. In the association study of *FOXP3* haplotypes, the following models were analyzed: A/C dominant (A/C carriers *versus* A/A, G/C, and G/A carriers), A/C recessive (ACAC *versus* A/A, G/C, and G/A carriers), A/A dominant (A/A carriers *versus* A/C, G/C, and G/A carriers), G/A dominant (G/A carriers *versus* A/C, A/A, and G/C carriers), G/A recessive (GAGA *versus* A/C, A/A, and G/C carriers), G/C dominant (G/C carriers *versus* A/C, A/A, and G/A carriers), and G/C recessive (GCGC *versus* A/C, A/A, and G/A carriers). The A/A recessive model (AAAA) was rare and was excluded from the analysis. The predominant haplotype was A/C (while the less frequent haplotype was A/A in our patient cohort.

Table 3 shows the distribution of *FOXP3* -924 G > A (rs2232365) and -3279 C > A (rs3761548) haplotypes among SLE patients and controls. We found an association between the A/A haplotype (dominant genetic model) with SLE (OR 3.729, 95% CI 1.006–13.820, *p* = 0.049) adjusted by age and ethnicity. On the other hand, we found a protective effect of the G/C haplotype (dominant genetic model) with SLE patients (OR 0.598, 95% CI 0.376–0.952, *p* = 0.030) adjusted by age and ethnicity.

**Table 3:** Distribution of *FOXP3* -924 G > A (rs2232365) and -3279 C > A (rs3761548) haplotype models among patients with systemic lupus erythematosus (SLE) and controls.

Haplotypes (rs2232365/rs3761548)	Controls (n = 157)	SLE (n = 193)	OR (95% CI)	*p* value*
A/C dominant	111 (71.6)	140 (72.5)	0.890 (0.546–1.453)	0.642
A/C recessive	29 (18.7)	31 (16.1)	0.889 (0.499–1.584)	0.690
A/A dominant	3 (1.9)	12 (6.2)	3.729 (1.006–13.820)	**0.049**
G/A dominant	84 (54.2)	106 (54.9)	1.115 (0.713–1.742)	0.633
G/A recessive	6 (3.9)	13 (6.7)	2.120 (0.755–5.951)	0.154
G/C dominant	67 (43.2)	70 (36.3)	0.598 (0.376–0.952)	**0.030**
G/C recessive	10 (6.5)	14 (7.3)	1.004 (0.414–2.434)	0.993

Bold values represent statistically significant values: OR (odds ratio) and CI (confidence interval) 95%. *Adjusted by age and ethnicity. Haplotype models: A/C dominant (A/C carriers versus A/A, G/C, and G/A carriers), A/C recessive (ACAC versus A/A, G/C, and G/A carriers), A/A dominant (A/A carriers versus A/C, G/A, and G/C carriers), G/A dominant (G/A carriers versus A/C, A/A, and G/C carriers), G/A recessive (GAGA versus A/C, A/A, and G/C carriers), G/C dominant (G/C carriers versus A/C, A/A, and G/A carriers), and G/C recessive (GCGC carriers versus A/C, A/A, and G/A carriers).

**Figure 1:**
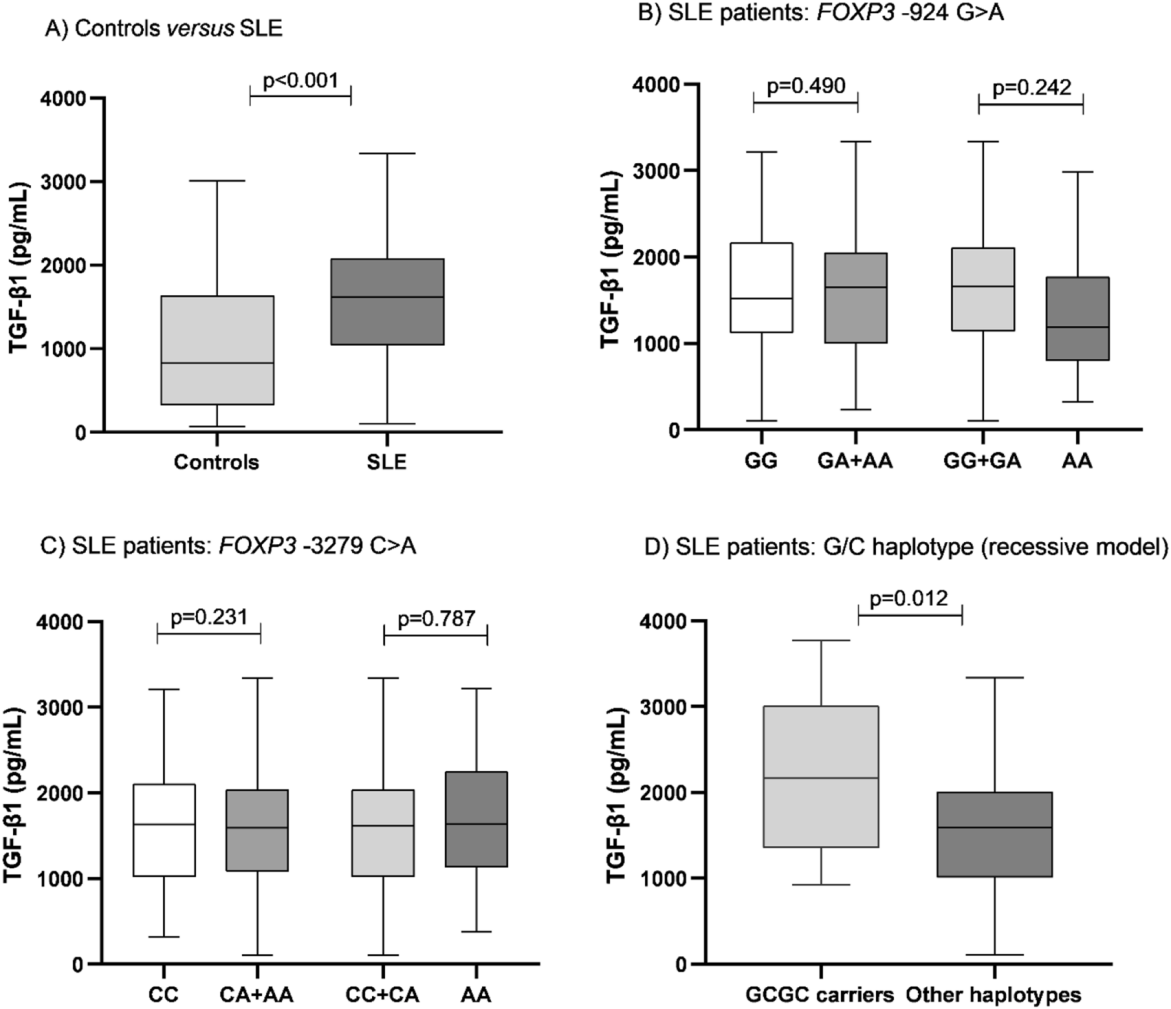
(**A**) Transforming growth factor (TGF)-β1 plasma levels in patients with systemic lupus erythematosus (SLE) and controls; (**B**) TGF-β1 plasma levels according to -924 G > A *FOXP3* variant (dominant and recessive model, respectively) in SLE patients; (**C**) TGF-β1 plasma levels according to -3279 C > A *FOXP3* variant (dominant and recessive model, respectively) in SLE patients; (**D**) TGF-β1 plasma levels according to the G/C recessive haplotype model in SLE patients. Results expressed as median and percentile (25–75). *p* value adjusted by age, ethnicity, and body mass index. G/C recessive haplotype model: GCGC carriers versus other haplotypes (A/C, A/A, and G/A carriers). Controls (n = 157), SLE (n = 196), SLE *FOXP3* -924 G > A: GG (n = 45), GA + AA (n = 148), GG + GA (n = 158), AA (n = 35), SLE *FOXP3* -3279 C > A: CC (n = 85), CA + AA (n = 111), CC + AA (n = 175), AA (n = 21), SLE GCGC haplotype (n = 14), other haplotypes (n = 179).

In the present study, we evaluated the TGF-β1 plasma levels in SLE patients and controls. Posteriorly, we evaluated these cytokine levels according to genotype and haplotype structure of *FOXP3* variants. SLE patients showed higher TGF-β1 plasma levels than controls (Fig. 1A) after adjusted by age, ethnicity, and BMI (*p* < 0.001). TGF-β1 plasma levels did not differ according to -924 G > A (Fig. 1B) and -3279 C > A (Fig. 1C) genotypes (dominant and recessive genetic models) in SLE patients, as well as among the controls (data not shown). However, SLE patients with the GCGC haplotype (G/C recessive model) had higher TGF-β1 plasma levels (*p* = 0.012) than other haplotypes (A/C, A/A or G/A carriers), after adjusted by age, ethnicity and BMI (Fig. 1D). In addition, TGF-β1 plasma levels did not differ in SLE patients according to other haplotype structures models (data not shown).

Furthermore, we analyzed whether the *FOXP3* variants (individually or in haplotype structure) could interfere in disease activity (C3, C4 and SLEDAI), the presence of autoantibodies and LN. These results are demonstrated in Table 4.

**Table 4:** Clinical and laboratory parameters of patients with systemic lupus erythematosus (SLE) according to *FOXP3* -924 G > A (rs2232365) and -3279 C > A (rs3761548) genotypes in dominant and recessive models.

SLE parameters	-924 G > A (dominant model)		-924 G > A (recessive model)		-3279 C > A (dominant model)		-3279 C > A (recessive model)	
	GG	GA + AA	GG + GA	AA	CC	CA + AA	CC + CA	AA
C3 (mg/dL)	115 (93–136)	111 (92–130)	115 (97–135)	106 (87–122)	107 (90–126)	116 (98–135)	112 (92–132)	115 (99–135)
C4 (mg/dL)	20.8 (13.4–26.2)	19.8 (13.6–25.7)	20.5 (13.5–26.0)	18.4 (13.3–23.2)	20.3 (14.1–27.7)	19.7 (13.4–25.8)	20.3 (13.6–25.8)	18.0 (13.5–26.5)
SLEDAI	2 (0–5)	4 (2–6)	3 (0–6)	2 (2–6)	2 (2–6)	2 (0–6)	4 (2–6)	2 (0–3)
SLEDAI ≥ 6	6 (13.6)	30 (20.5)	30 (19.2)	6 (17.6)	13 (15.7)	23 (20.9)	33 (19.2)	3 (14.3)
Anti-nucleosome (IU/mL)	38.1 (15.7–125.0)	58.3 (24.5–141.0)	52.4 (19.4–133.0)	77.2 (24.5–176.0)	65.7 (20.2–159.0)	44.8 (17.8–131.0)	51.3 (18.6–132.0)	97.0 (30.6–315.0)
Anti-dsDNA Positive	20 (47.6)	86 (61.4)	81 (54.0)	25 (78.1)*	48 (61.5)	59 (55.7)	96 (58.5)	11 (55.0)
Anti-SM Positive	5 (16.1)	25 (25.3)	23 (20.9)	7 (35.0)	17 (31.5)	13 (17.1)	26 (22.4)	4 (28.6)
Anti-U1RNP Positive	14 (50.0)	57 (58.8)	43(40.6)	11(57.9)*	26 (49.1)	28 (38.9)	49 (43.4)	5 (41.7)
Lupus nephritis	21 (48.8)	64 (45.4)	18 (54.5)	67 (44.4)	45 (56.2)	42 (39.3)*	78 (46.7)	9 (45.0)

Data were expressed by median and percentile (25–75) or absolute number (n) and percentage (%). * p < 0.05, adjusted by age, ethnicity, body mass index and treatment. C3: complement 3; C4: complement 4; SLEDAI: systemic lupus erythematosus disease activity index; Anti-dsDNA: anti-double-stranded DNA; Anti-SM; anti-Smith; Anti-U1RNP: anti-U1 ribonucleoprotein.

Patients with -924 AA genotype (recessive genetic model) showed higher frequency of anti-dsDNA (*p* = 0.012) and anti-U1RNP (*p* = 0.036) antibodies, even after adjusted by age, ethnicity, BMI, and treatment. However, the genotypes did not differ regarding the parameters of disease activity and frequency of nephritis (*p* > 0.05). Regarding the genetic variant of *FOXP3* -3279 C > A (rs3761548), there was no association with autoantibodies and disease activity. However, patients with CA + AA genotype (dominant genetic model) had lower frequency of nephritis (*p* = 0.038), adjusted by age, ethnicity, BMI and treatment.

Table 5 showed *FOXP3* haplotype structures and SLE parameters. Patients with A/C haplotype (dominant genetic model) had higher SLEDAI score [OR 1.119, CI 95% (1.015–1.234), *p* = 0.024] while those with the same haplotype, but in the recessive model (ACAC), had higher frequency of anti-dsDNA positivity [OR 3.026, CI 95% (1.062–8.624), *p* = 0.038], anti-U1RNP positivity [OR 5.649, CI 95% (1.199–26.610), *p* = 0.029], and nephritis [OR 2.501, CI 95% (1.004–6.229), *p* = 0.049]. In addition, SLE patients with A/A haplotype (dominant genetic model) had higher levels of anti-nucleosome antibodies [OR 1.004, CI 95% (1.001–1.008), *p* = 0.026].

**Table 5:** Clinical and laboratory parameters of patients with systemic lupus erythematosus (SLE) according to *FOXP3* -924 G > A (rs2232365) and -3279 C > A (rs3761548) haplotype models.

SLE parameters	A/C dominant	A/C recessive	A/A dominant	G/A dominant	G/A recessive	G/C dominant	G/C recessive
	*p* value	*p* value	*p* value	*p* value	*p* value	*p* value	*p* value
C3 (mg/dL)	0.557	0.749	0.717	0.390	0.922	0.496	0.607
C4 (mg/dL)	0.896	0.599	0.640	0.503	0.964	0.164	0.819
SLEDAI	**0.024**^**1**^	0.439	0.954	0.339	0.211	0.583	0.344
SLEDAI ≥ 6	0.297	0.138	0.111	0.908	0.680	0.731	0.235
Anti-nucleosome (IU/mL)	0.312	0.854	**0.026**^**5**^	0.513	0.776	0.433	0.939
Anti-dsDNA Positive	0.119	**0.038**^**2**^	0.507	0.097	0.620	0.179	0.924
Anti-SM Positive	0.662	0.080	0.324	0.082	0.547	0.935	0.855
Anti-U1RNP Positive	0.189	**0.029**^**3**^	0320	0.346	0.251	0.907	0.245
Lupus nephritis	0.251	**0.049**^**4**^	0.062	0.161	0.750	0.381	0.301

Bold values represent statistically significant values. *p value adjusted by age, ethnicity, body mass index, and treatment. C3: complement 3; C4: complement 4; SLEDAI: systemic lupus erythematosus disease activity index; Anti-dsDNA: anti-double-stranded DNA; Anti-SM; anti-Smith; Anti-U1RNP: anti-U1 ribonucleoprotein. Haplotype models: A/C dominant (A/C carriers versus A/A, G/C, and G/A carriers), A/C recessive (ACAC versus A/A, G/C, and G/A carriers), A/A dominant (A/A carriers versus A/C, G/A, and G/C carriers), G/A dominant (G/A carriers versus A/C, A/A, and G/C carriers), G/A recessive (GAGA versus A/C, A/A, and G/C carriers), G/C dominant (G/C carriers versus A/C, A/A, and G/A carriers), and G/C recessive (GCGC carriers versus A/C, A/A, and G/A carriers). ^1^ OR 1.119, CI 95% (1.015–1.234), *p* = 0.024; ^2^ OR 3.026, CI 95% (1.062–8.624), *p* = 0.038; ^3^ OR 5.649, CI 95% (1.199–26.610), *p* = 0.029; ^4^ OR 2.501, CI 95% (1.004–6.229), *p* = 0.049; ^5^ OR 1.004, CI 95% (1.001–1.008), *p* = 0.026.

## Discussion

The main findings of the present study were that the AA genotype of *FOXP3* -3279 C > A (rs3761548) was associated with a 2.6-fold chance of developing SLE than other genotypes. Also, we found an association between the *FOXP3* haplotype structures (rs2232365/ rs3761548) and SLE susceptibility. A/A haplotype (dominant genetic model) was associated with a 3.7-fold chance to develop SLE. On the other hand, the G/C haplotype (dominant genetic model) showed a protective effect of 40.0% in the susceptibility to SLE. Moreover, patients with GCGC haplotype had higher levels of TGF-β1. In addition, we demonstrated that *FOXP3* variants could interfere in SLE parameters. Patients with A/C haplotype in the dominant model had a higher SLEDAI score, and patients with ACAC haplotype had a threefold and 5.6-fold chance to have anti-dsDNA and anti-U1RNP positive, respectively, and 2.5-fold higher susceptibility to nephritis.

*FOXP3* was initially identified as a gene responsible for X-linked autoimmune diseases in humans and a master regulator of the development and function of Treg ^34^. Mainly expressed in CD4^+^CD25^+^Treg cells, *FOXP3* encodes a transcriptional factor that is involved in T cells activation and its expression is essential for driving CD4^+^CD25^+^FOXP3^+^Treg cells function as suppressor T cells^35,36^. Previous studies demonstrated that alteration of *FOXP3* expression and functions could contribute to various autoimmune diseases due to a functional block of Treg cells^31^.

Previously, our group evaluated the -3279 C > A of *FOXP3* variant (rs3761548) and demonstrated that the presence of the A allele increased the chance to have multiple sclerosis diagnosis in female patients^22^. The presence of the A allele of *FOXP3* -3279 alters the promoter region and consequently, there is a loss of binding of some transcription factors, such as E47 and C-Myb, leading to defective transcription of *FOXP3*^37^, and therefore, might affect the function or quantity of Tregs^38^. In the present study, we demonstrated that the A allele of *FOXP3* -3279 C > A (rs3761548), in homozygosis or heterozygosis, confers 2.6-fold chance of SLE diagnosis. Until now, only a previous study evaluated this variant in SLE patients and did not find any association with SLE susceptibility^8^. Discrepancies in the allelic/genotypes frequencies between studies could be explained by the heterogeneity of the studied diseases, ethnicity, the limited sample size, as well as the method of genotyping and the characteristics of the control group^31^.

Regarding the -924 G > A (rs2232365), we did not find any association of this variant and SLE susceptibility and clinical parameters. Although the -924 G > A *FOXP3* variant (rs2232365) was evaluated in other autoimmunity diseases^23–25^ this is the first study to evaluate this variant in SLE patients. The G > A substitution of *FOXP3* -924 is located in a putative-binding site for the transcription factor GATA-3^39^. This transcription factor binds to the promoter region of *FOXP3* to inhibits its expression only when the A allele is present. To occur *FOXP3* expression, GATA-3 must be removed from the promoter region^40^. So, GG carriers lose their GATA-3-binding site, enabling *FOXP3* gene transcription.

Genetic variants do not exert great influence by itself^41^ and the analysis in combination is better to understand the role of *FOXP3* variants in SLE. Thus, we investigated the haplotype structures of *FOXP3* -924 G > A (rs2232365) and -3279 C > A (rs3761548) variants. The G/C haplotype (dominant genetic model) showed a protective effect of 40.0% in the susceptibility to SLE. While the A/A haplotype (dominant genetic model) demonstrated to be associated with SLE susceptibility and the heritance of at least one A allele of each variant increases lupus susceptibility to 3.7 times.

In the present study, we investigated the influence of *FOXP3* variants in TGF-β1 plasma levels, a multifunctional cytokine with immunomodulatory effects. Initially, we found higher TGF-β1 plasma levels in SLE patients compared to control group. Therefore, we hypothesized that the increased TGF-β1 plasma levels, probably, could represent an endogenous anti-inflammatory response aimed at counteracting ongoing immunoinflammatory events in the SLE patients. In addition, our data demonstrated that *FOXP3* -924 G > A and -3279 C > A genotypes individually were not associated with TGF-β1 plasma levels in SLE patients. However, patients with the GCGC haplotype showed higher TGF-β1 plasma levels compared to other haplotype structures and could explain the protect effect showed by G/C haplotype. This is the first study to evaluate the association of these SNVs of *FOXP3* with cytokines levels in SLE patients.

Regarding *FOXP3* variants and SLE parameters, we found that the -924 AA genotype was associated with anti-dsDNA and anti-U1RNP antibodies positivity, independently of extraneous factors (age, ethnicity and BMI). However, we failed to demonstrate association between the -3279 C > A and autoantibodies. Our data disagreed with a previous study that showed patients carrying the -3279 C allele had higher anti-dsDNA levels^8^. However, our patients with the ACAC haplotype had a threefold chance to have anti-dsDNA positivity, 5.6-fold chance to have anti-U1RNP positivity, and 2.5-fold chance to have nephritis. In addition, we demonstrated that SLE patients carrying the A allele of -924 G > A and the C allele of -3279 (A/C haplotype in dominant model) had higher SLEDAI score than those with other haplotype combinations. Antibodies to dsDNA are usually present at high titers in SLE patients with active nephritis^8^. Thus, it seems reasonable to hypothesize that the presence of the A allele, from both *FOXP3* variants, could favor autoimmunity, activity disease and development of nephritis. Although we identified that haplotypes of the abovementioned *FOXP3* variants were associated to the antibody production and pathogenesis of lupus nephritis, the mechanisms by which this may occur needs to be elucidate. More specific studies on the functional role of this gene in SLE, will be necessary.

Some limitations of this study should be considered. This is a case–control design, which does not allow inferences on causal relationship. In addition, most SLE patients had inactive or mild disease activity, and parameters such as anti-dsDNA, cytokines, and complement levels, fluctuate significantly during the course of SLE. This is a major limitation of such association studies. However, blood samples and laboratory analyzes were performed at the time of inclusion in the study, demonstrating the disease profile in that specific moment. The study also has some strengths, such as the robust statistical analysis, with adjusting for some confounding variables including age, ethnicity, BMI, and treatment. In addition, this is the first study to investigate the *FOXP3* -924 G > A (rs2232365) and *-*3279 C > A (rs3761548) variants, individually and haplotype, in SLE female patients.

In conclusion, the heritance of at least one A allele of each variant (rs2232365/ rs3761548) increases SLE susceptibility while patients with A/C haplotype in the dominant model had a higher SLEDAI score and patients with ACAC haplotype structure are associated with anti-dsDNA and anti-U1RNP antibodies, and higher susceptibility to nephritis. Furthermore, patients with the GCGC haplotype showed higher TGF-β1 plasma levels and G/C haplotype in the dominant model showed a protective effect in SLE susceptibility.

Our data demonstrate that the genetic variants of *FOXP3* are associated with SLE susceptibility. The G/C haplotype provides protection for SLE, possibly by increasing TGF-β levels. While the presence of allele A of both variants, could favor autoimmunity, disease activity and presence of LN. The impact of these genetic variants in the immunity imbalance and their relation to autoantibodies and disease activity lead to significant information regarding the role of *FOXP3* in SLE pathophysiology.
